# Monthly Record of Deaths in the City of Buffalo

**Published:** 1855-02

**Authors:** J. M. Newman

**Affiliations:** Health Physician


ART. X.—*Record of Mortality in the City of Buffalo, for the Month of December*, 1854. By J. M. NEWMAN, M. D., Health Physician.

| DISEASES. | No. | Males. | Females. |
|---|---|---|---|
| Abscessus Recti, | 1 | 1 | |
| Accidens, | 1 | 1 | |
| Anæmia, | 1 | | 1 |
| Apoplexia, | 1 | 1 | |
| Asthma, | 1 | 1 | |
| Bronchitis, | 2 | 2 | |
| Cancer Pulmonalis, | 1 | 1 | |
| Capite Punitus, | 1 | 1 | |
| Cynanche Trachealis, | 3 | | 3 |
| Debilitas, | 10 | 3 | 3 |
| Diarrhœa, | 1 | | 1 |
| Dysenteria, | 1 | 1 | |
| Eclampsia, | 11 | 6 | 4 |
| Febris Scarlatina, | 10 | 2 | 8 |
| " Typhoid, | 5 | 4 | 1 |
| " Typhus, | 1 | | 1 |
| Hæmorrhagia Uterina, | 1 | | 1 |
| " Pulmonis, | 1 | 1 | |
| Hydrops Cerebri, | 6 | 3 | 2 |
| Hyperæmia Cordis, | 1 | | 1 |
| Gangræna Pulmonium, | 1 | 1 | |
| Intemperantia et. Delir. Tremens, | 5 | 3 | 2 |
| Marasmus, | 2 | | 2 |
| Mortificatio Genu, | 1 | 1 | |
| Natus Mortuus, | 3 | | |
| Negligentia, | 1 | | 1 |
| Parturitio, | 2 | | 2 |
| Pertussis, | 1 | | 1 |
| Peritonitis, | 1 | 1 | |
| Phrenitis, | 3 | 2 | 1 |
| Phthisis Pulmonalis, | 25 | 10 | 14 |
| Pneumonia, | 2 | 1 | 1 |
| " Typhoid, | 1 | 1 | |
| Rubecla, | 1 | 1 | |
| Senectus, | 8 | 4 | 4 |
| Tumeur Ovarium, | 1 | | 1 |
| Ignotus, | 18 | 13 | 1 |
| | 136 | | |

| | |
|---|---|
| Males, | 66 |
| Females, | 56 |
| Sex not given, | 14 |
| Total, | 136 |

REGISTER OF MORTALITY—(Continued.)

AGES.

| | | | |
|---|---|---|---|
| Still-born, | 3 | Between 6 years and 12 years, | 4 |
| 1 day; | 3 | " 12 " " 20 " | 7 |
| Between 1 day and 15 days, | 8 | " 20 " " 30 " | 15 |
| " 15 days and 30 days, | 5 | " 30 " " 40 " | 19 |
| " 1 month and 3 months, | 8 | " 40 " " 50 " | 12 |
| " 3 months and 6 months, | 4 | " 50 " " 60 " | 3 |
| " 6 " " 9 " | 3 | " 60 " " 70 " | 2 |
| " 9 " " 12 " | 5 | " 70 " " 80 " | 6 |
| " 1 year and 3 years, | 20 | " 80 " " 90 " | 2 |
| " 3 years and 6 years, | 7 | " 90 " " 100 " | 0 |
| Total, | | | 136 |

NATIVITIES.

| | | | |
|---|---|---|---|
| American, | 61 | Belgian, | 0 |
| English, | 5 | French, | 3 |
| Scotch, | 1 | Swiss, | 2 |
| Irish, | 16 | Hollander, | 3 |
| Canadian, | 0 | Colored, | 0 |
| German, | 33 | Country not given, | 12 |
| Total, | | | 136 |

